# The association of body mass index with the risk of type 2 diabetes: a case–control study nested in an electronic health records system in the United States

**DOI:** 10.1186/1758-5996-6-50

**Published:** 2014-04-03

**Authors:** Michael L Ganz, Neil Wintfeld, Qian Li, Veronica Alas, Jakob Langer, Mette Hammer

**Affiliations:** 1 Evidera, 430 Bedford Street, Lexington, MA 02420, USA; 2 Novo Nordisk, Inc., 800 Scudders Mill Road, Plainsboro, New Jersey 08536, USA

**Keywords:** Obesity, Risk of type 2 diabetes, Electronic health records

## Abstract

**Objectives:**

Obesity is a known risk factor for type 2 diabetes (T2D). We conducted a case–control study to assess the association between body mass index (BMI) and the risk of being diagnosed with T2D in the United States.

**Methods:**

We selected adults (≥ 18 years old) who were diagnosed with T2D (defined by ICD-9-CM diagnosis codes or use of anti-diabetic medications) between January 2004 and October 2011 (“cases”) from an electronic health records database provided by an integrated health system in the Middle Atlantic region. Twice as many individuals enrolled in the health system without a T2D diagnosis during the study period (“controls”) were selected based on age, sex, history of cardiac comorbidities or hyperinflammatory state (defined by C-reactive protein and erythrocyte sedimentation rate), and use of psychiatric or beta blocker medications. BMI was measured during one year prior to the first observed T2D diagnosis (for cases) or a randomly assigned date (for controls); individuals with no BMI measure or BMI < 18.5 kg/m^2^ were excluded. We assessed the impact of increased BMI (overweight: 25–29.9 kg/m^2^; Obesity Class I: 30–34.9 kg/m^2^; Obesity Class II: 35–39.9 kg/m^2^; Obesity Class III: ≥40 kg/m^2^), relative to normal BMI (18.5–24.9 kg/m^2^), on a T2D diagnosis using odds ratios (OR) and relative risks (RR) estimated from multiple logistic regression results.

**Results:**

We included 12,179 cases (mean age: 55, 43% male) and 25,177 controls (mean age: 56, 45% male). We found a positive association between BMI and the risk of a T2D diagnosis. The strength of this association increased with BMI category (RR [95% confidence interval]: overweight, 1.5 [1.4–1.6]; Obesity Class I, 2.5 [2.3–2.6]; Obesity Class II, 3.6 [3.4–3.8]; Obesity Class III, 5.1 [4.7–5.5]).

**Conclusions:**

BMI is strongly and independently associated with the risk of being diagnosed with T2D. The incremental association of BMI category on the risk of T2D is stronger for people with a higher BMI relative to people with a lower BMI.

## Background

Diabetes, the most common metabolic disorder, is associated with substantial disease burden, including increased mortality risk and significant long-term morbidity [1-4]. Diabetes was diagnosed in 22.3 million people (7% of the population) in the United States (US) in 2012 and was responsible for $176 billion of direct medical costs and $69 billion of indirect (lost productivity) costs [5]. Type 2 diabetes mellitus (T2D) comprises about 90%–95% of all diabetes cases [6,7], and its prevalence has been steadily increasing [8]. Obesity, classified as body mass index (BMI) ≥30 kg/m^2^, is a known predictor of T2D and has become a major public health problem in the US [9], affecting over one-third (35.7%) of the population [10]. It costs about $190.2 billion (in 2005 dollars) annually to treat obesity for the non-institutionalized US adult population, which accounts for almost 21% of US healthcare expenditures [11]. Healthcare costs attributable to obesity and overweight in the US are projected to reach $860.7 billion by 2030 [12].

The impact of BMI classification, including overweight and various grades of obesity, on the risk of T2D in the real-world practice is a well-investigated topic [9,13]. However, the evidence for the US is dated, with most of the studies relying on data before the year 2005 and does not reflect recent changes in the obesity ‘epidemic’ [14-21]. We designed this case–control study to obtain more recent evidence of the association between BMI and the risk of being diagnosed with T2D in US.

## Materials and methods

### Data and Study Sample

Data were obtained from the MedMining® database, which contains electronic health records from the Geisinger Health System. The Geisinger Health System, which serves more than 4 million individuals in the state of Pennsylvania, is an integrated health system with an 880+ multi-specialty physician group practice, 5 hospital campuses, 72 primary and specialty clinic sites, and a health plan. Individuals’ health records, which have been kept in electronic form at Geisinger Health System since 1996, contain information on demographic characteristics (age, sex, and race/ethnicity), encounter details from inpatient, outpatient, and office-based settings (such as ICD-9-CM diagnosis codes, and CPT-4 procedure codes), medication orders, lab findings, and actual costs incurred by the Geisinger Health System for those encounters. This dataset has been widely used to address the health economic evaluations in real-world settings.[22-26]

Cases were selected if their first diagnosis of T2D (defined by ICD-9-CM diagnosis codes 250.x0 or 250.x2 or by an anti-diabetic medication order, whichever came first) while in the Geisinger Health System database was observed between January 2004 and October 2011 (study period). Events and measurements were anchored by each individual’s “index date,” which was defined as the date of their incident, or first observed, T2D diagnosis in the MedMining database.

We used ICD-9-CM diagnosis codes and laboratory values to measure the history of any cardiac comorbidities (lipid abnormalities, coronary heart disease, acute myocardial infarction, angina pectoris, or hypertension), hyperinflammatory state (defined by clinical biomarkers of C-reactive protein and erythrocyte sedimentation rate), psychiatric medication use (anticonvulsants or antipsychotic medication), and beta-blocker medication use during each case individual’s 12-month pre-index period.

We created a group of potential controls by randomly selecting two individuals with no history of diabetes (defined by ICD-9-CM diagnosis codes 250.xx or use of any anti-diabetic medication) during the study period for each case. Cases and potential controls were selected based on age group (< 65 or ≥ 65), sex, and history of any cardiac comorbidities, hyperinflammatory state, psychiatric medication use, and beta-blocker medication use. Since the potential controls never received a T2D diagnosis, we assigned them a random index date between the beginning and end of the study period. Individuals (both cases and potential controls) were further required to be alive as of October 31, 2011, be ≥18 years old at index date, and satisfy all of the selection criteria listed in the Appendix. Baseline BMI was defined as the last value observed during the 12-month pre-index period for both cases and controls, and was classified according to the World Health Organization’s definition of BMI: normal (18.5–24.9 kg/m^2^), overweight (25–29.9 kg/m^2^), Obesity Class I (30–34.9 kg/m^2^), Obesity Class II (35–39.9 kg/m^2^), and Obesity Class III (≥40 kg/m^2^).

### Analysis

We compared individual demographic and clinical characteristics, including baseline BMI, between cases and controls and assessed the statistical significance (p < 0.05) of the differences between groups using the Student’s t-test for continuous variables and the chi-square test for categorical variables.

We assessed the impact of baseline BMI on the risk of T2D diagnosis via the odds ratios (ORs) estimated from an unconditional multiple logistic regression model that adjusted for other covariates such as the index year, smoking status, employment status, payer status, Geisinger Health Plan coverage, history of depression, use of selected medications (to treat depression/anxiety, obesity, hyperlipidemia, and hypertension) and medical burden during the 12-month pre-index period. Medical burden was measured by any use of outpatient, inpatient, or emergency services, as well as the logarithm of the total annual encounter costs adjusted to 2011 dollars using the US Consumer-Price Index [27]. Age group was also included to address the fact that the age variable used in the matching procedure was categorical (≥ 65 years or not). In addition to the adjusted ORs, we estimated the adjusted relative risk of T2D diagnosis for each BMI category (with normal BMI as the comparator) using the method of recycled predictions [28]; 95% confidence intervals (CIs) around the relative risks were estimated by the 2.5 and 97.5 percentiles of 1,000 bootstrap replications [29]. Because we sampled cases and controls independent of exposure status (i.e., BMI), which is consistent with the case-cohort sampling approach, these relative risk estimates are applicable to the whole Geisinger Health System patient population [30]. Data were compiled and analyzed using SAS (version 9.2, SAS Institute Inc., Cary, NC).

## Results

**Figure 1:**
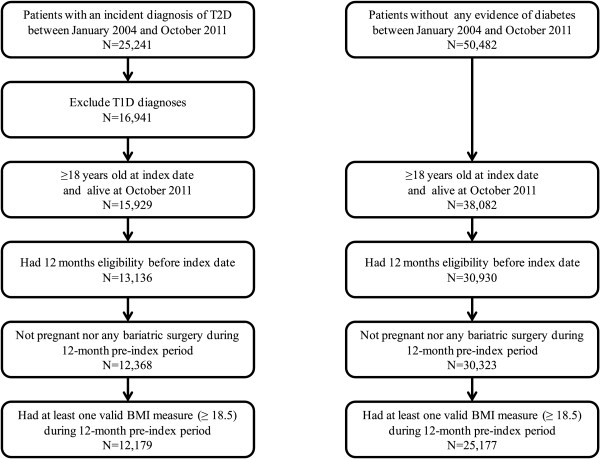
Sample Selection.

We identified 25,241 individuals who experienced their incident T2D diagnosis between January, 2004 and October, 2011, and 50,482 matched control individuals with no history of diabetes during the same time frame. The final sample included 37,356 individuals who had a baseline BMI ≥18.5 kg/m^2^ (12,179 cases and 25,177 controls) after we applied the sample selection criteria (Figure 1). Table 1 displays the individuals’ demographic and clinical characteristics. Compared with control individuals, the case individuals had higher baseline BMI values (mean ± standard deviation: 35.4 ± 8.5 kg/m^2^ vs. 29.4 ± 6.3 kg/m^2^, p < 0.01). Cases were more likely to be younger, male, and to have higher healthcare resource use as measured by costs during the 12-month pre-index period than controls. Cases were also more likely to have experienced comorbidities related to diabetes and/or obesity and used medications related to diabetes or obesity during the 12-month pre-index period than controls.

**Table 1:** Demographic and Clinical Characteristics of the Analytic Sample

	**Cases**	**Controls**	**P value**
	**(With T2D) N = 12,179**	**(Without diabetes) N = 25,177**	
BMI (mean ± SD, kg/m^2^)	35.4 ± 8.5	29.4 ± 6.3	<0.01
BMI category (%)			<0.01
18.5–24.9 kg/m^2^	7.4	24.1	
25.0–29.9 kg/m^2^	20.5	36.1	
30.0–34.9 kg/m^2^	26.3	23.3	
35.0–39.9 kg/m^2^	20.6	10.0	
40+ kg/m^2^	25.3	6.5	
Age (mean ± SD)	55 ± 15.9	56 ± 18.2	<0.01
Age group (%)			<0.01
18.5–24.9	25.0	27.6	
25.0–29.9	45.2	34.2	
30.0–34.9	17.5	20.8	
35.0–39.9	10.3	13.7	
40+	2.0	3.7	
Male (%)	42.9	44.6	<0.01
Race (%)			<0.01
White	97.1	97.5	
Black	1.8	1.3	
Other/unknown	1.1	1.2	
Index year (%)			<0.01
2005	8.8	10.0	
2006	13.0	10.9	
2007	15.3	12.2	
2008	15.5	14.1	
2009	18.9	16.2	
2010	16.4	18.8	
2011	12.0	17.8	
Smoking status (%)			<0.01
Never smoke	49.0	48.0	
Former smoker	29.8	22.6	
Current smoker	17.8	15.4	
Other/unknown	3.4	14.0	
Employment status (%)			<0.01
Full Time	37.1	32.4	
Not employed	22.2	15.5	
Other/unknown	40.8	52.1	
Payer status (%)			<0.01
Commercial	78.9	72.4	
Medicare	12.8	14.1	
Medicaid	4.6	3.1	
Other/unknown	3.6	10.4	
Covered by Geisinger Health Plan (%)	49.7	43.8	<0.01
Any cardiac comorbidities (%)	85.3	63.1	<0.01
Hyperinflammatory state (%)	6.3	2.1	<0.01
Depression (%)	8.2	4.1	<0.01
Psychiatric drugs (%)	14.7	7.7	<0.01
Antidepressants/anxiolytics (%)	36.5	22.4	<0.01
Anti-obesity drugs (%)	0.4	0.1	<0.01
Beta blockers (%)	32.3	21.4	<0.01
Antihyperlipidemia drugs (%)	36.7	23.1	<0.01
Antihypertensives (%)	35.2	21.1	<0.01
Any outpatient encounters (%)	95.8	88.3	<0.01
Any inpatient encounters (%)	16.6	7.9	<0.01
Any emergency department encounters (%)	14.3	8.8	<0.01
Total encounter costs ($,mean ± SD)	6,324 ± 17,253	2,640 ± 7,606	<0.01

Data source: Geisinger Health System electronic health records, January 2004–October 2011.

Characteristics were measured at index date or during 1 year before index date.

Any cardiac comorbidities: lipid abnormalities, coronary heart disease, acute myocardial infarction, angina pectoris, and hypertension, were defined by both ICD-9-CM diagnosis codes and clinical biomarkers.

Hyperinflammatory state: defined by clinical biomarkers of C-reactive protein and erythrocyte sedimentation rate.

Psychiatric medication use: anticonvulsants or antipsychotic medication.

Total encounter costs: in 2011 dollars.

P value based on t-test for continuous variables and chi-squared test for categorical variables.

**Figure 2:**
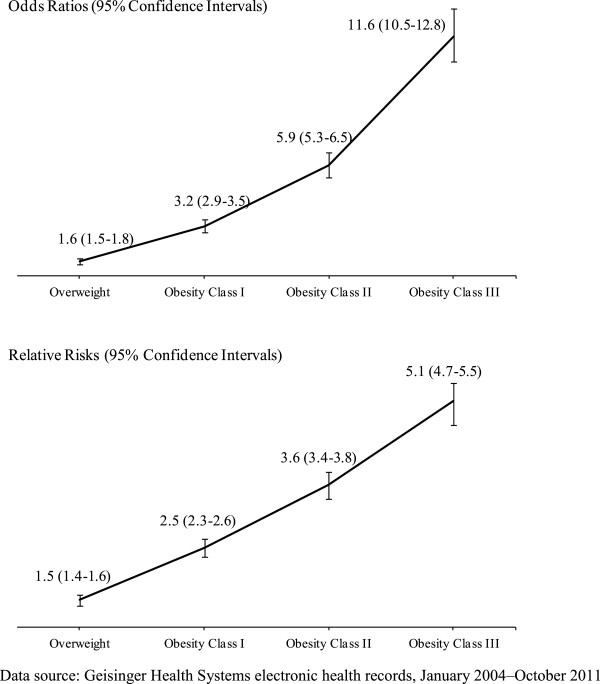
**Odds Ratios and Relative Risks of T2D Diagnosis, by BMI Categories**. Data source: Geisinger Health System electronic health records, January 2004–October 2011. Odds ratios and 95% confidence intervals (CI) were estimated using logistic regression, adjusted for baseline demographic and clinical characteristics. Relative risks were estimating using the method of recycled predictions and 95% CIs were estimated from bootstrap replications. BMI categories: normal: 18.5–24.9 kg/m^2^; overweight: 25–29.9 kg/m^2^; Obesity Class I: 30–34.9 kg/m^2^; Obesity Class II: 35–39.9 kg/m^2^; Obesity Class III: ≥40 kg/m^2^.

As shown in the first 4 rows of Table 2 and in the upper panel of Figure 2, compared with individuals with a normal BMI, individuals who were overweight or obese were more likely to be diagnosed with T2D (OR [95%CI]: ranging from 1.6 [1.5–1.8] for overweight adults to 11.6 [10.5–12.8] for adults in Obesity Class III, all p-values < 0.01). The relative risks displayed a similar pattern: the relative risk was 1.5 (95%CI: 1.4–1.6) for overweight adults, 2.5 (2.3–2.6) for adults in Obesity Class I, 3.6 (3.4–3.8) for adults in Obesity Class II, and 5.1 (4.7–5.5) for adults in Obesity Class III (lower panel of Figure 2).

**Table 2:** Logistic Regression for Risk of T2D Diagnosis

	**Odds Ratio**	**95% CI**
BMI category (reference: 18.5–24.9 kg/m^2^)		
25.0–29.9 kg/m^2^	1.63	1.49–1.78
30.0–34.9 kg/m^2^	3.19	2.92–3.48
35.0–39.9 kg/m^2^	5.86	5.32–6.46
40+ kg/m^2^	11.58	10.46–12.82
Age group (reference: 18–44)		
45–64	1.19	1.11–1.28
65–74	0.74	0.67–0.81
75–84	0.79	0.70–0.88
85+	0.75	0.63–0.89
Male	0.97	0.92–1.02
Race (reference: White)		
Black	1.44	1.18–1.75
Other/unknown	1.58	1.25–2.00
Index year (reference: 2005)		
2006	1.35	1.21–1.50
2007	1.44	1.30–1.60
2008	1.22	1.10–1.35
2009	1.30	1.17–1.43
2010	0.95	0.85–1.04
2011	0.65	0.58–0.72
Smoking status (reference: Never smoke)		
Former smoker	1.18	1.11–1.25
Current smoker	1.15	1.07–1.23
Other/unknown	0.40	0.35–0.45
Employment status (reference: Full time)		
Not employed	1.14	1.06–1.23
Other/unknown	1.08	1.01–1.16
Payer status (reference: Commercial)		
Medicare	1.13	1.04–1.23
Medicaid	1.11	0.97–1.27
Other/unknown	0.92	0.81–1.05
Covered by Geisinger Health Plan	1.23	1.16–1.29
Any cardiac comorbidities	1.70	1.58–1.83
Hyperinflammatory state	2.08	1.83–2.37
Depression	1.14	1.02–1.27
Psychiatric drug	1.31	1.21–1.43
Antidepressants/anxiolytics	1.19	1.12–1.26
Anti-obesity drug	1.39	0.81–2.38
Beta blocker	1.04	0.98–1.10
Antihyperlipidemia drugs	1.38	1.30–1.47
Antihypertensives	1.32	1.24–1.40
Any outpatient encounters	0.66	0.57–0.76
Any inpatient encounters	1.31	1.19–1.43
Any emergency department encounters	1.03	0.95–1.12
Log of total encounter costs	1.17	1.14–1.19
C statistic = 0.79		

Data source: Geisinger Health System electronic health records, January 2004–October 2011.

Characteristics were measured at index date or during 1 year before index date.

Any cardiac comorbidities: lipid abnormalities, coronary heart disease, acute myocardial infarction, angina pectoris, and hypertension.

Hyperinflammatory state: defined by C-reactive protein and erythrocyte sedimentation rate.

Psychiatric medication use: anticonvulsants or antipsychotic medication.

Total encounter costs: in 2011 dollars.

Furthermore, we found that the change in the magnitude of the ORs from one BMI category to the next was larger for individuals in higher BMI categories than individuals in lower BMI categories, as illustrated by the increasing slope of the lines connecting the ORs and, to a lesser degree, the lines connecting the relative risks in Figure 2. These patterns of ORs and relative risks imply that individuals in higher BMI categories were increasingly more likely to be diagnosed with T2D than individuals in lower BMI categories (p < 0.05).

Other individual characteristics, aside from BMI, were also significantly associated with the risk of being diagnosed with T2D. Individuals who were 45–64 years old (compared with 18–44 years old), were black or other race (compared with white), or ever smoked (compared with never) were associated with an increased risk of T2D diagnosis. In addition, individuals who experienced comorbidities (any cardiac comorbidities, hyperinflammatory state, or depression) or who used medications (psychiatric drugs, antidepressants or anxiolytics, antihyperlipidemia drugs, and antihypertensives) were more likely to have been diagnosed with T2D than those who did not, as were individuals with more medical costs in the pre-index period. However, the ORs of the individual characteristics (except for BMI) were not the focus of this study since they were included in the regression in order to adjust for the impact of BMI.

## Discussion

After adjusting for a number of characteristics associated with the risk of T2D, we found that, compared with normal BMI, overweight and obesity was statistically significantly associated with the risk of being diagnosed with T2D among individuals without any other prior evidence of T2D. We further found that the risk of a T2D diagnosis was increasingly larger for individuals in higher BMI categories than for individuals in lower BMI categories.

Our results are consistent with other studies that have examined the association between BMI and risk of T2D using nationally representative samples. For example, using data from the 2001 Behavioral Risk Factor Surveillance System, Mokdad et al. (2003) also found statistically significant and increasingly larger ORs for T2D among overweight adults (1.59, 95% CI: 1.46–1.73), adults with BMI between 30 and 39.9 kg/m^2^ (3.44, 95% CI: 3.17–3.74), and adults with BMI ≥ 40 kg/m^2^ (7.37, 95% CI: 6.39–8.50) relative to adults with normal BMI [20]. According to the Nurses’ Health Study, the adjusted relative risk of T2D associated with each 5-unit increment in BMI ranged from 1.55 (95% CI: 1.36–1.77) to 2.36 (95% CI: 1.83–3.04) among women, depending on the participants’ race/ethnicity, in the 1980–2000 prospective cohort [15]; and the overall relative risk of non-insulin-dependent T2D among women with BMI ≥ 29.9 kg/m^2^ relative to women with BMI ≤ 20.1 kg/m^2^ in the 1986–1994 cohort was 11.2 (95% CI: 7.9–15.9) [14]. Although regional data were used, the current study covered more recent years.

Moreover, BMI values were clinically measured in the current study, compared with BMI calculated from self-reported height and weight in those earlier studies. Self-reported weight and height considerably underestimate the individuals’ measured BMI [31,32] and may thus have weakened the association between obesity and risk of T2D and/or biased the estimated results. This may explain the lower ORs associated with BMI levels in the Mokdad et al. study, compared to the current study.

Our results are also consistent with the studies that investigated the association between BMI and risk of T2D among individuals with pre-diabetes. The Diabetes Prevention Program (DPP) is a large randomized clinical trial that ran from 1996 to 2001 (average follow-up: 2.8 years) and that enrolled individuals at higher risk for T2D (all subjects had impaired glucose tolerance at baseline) [33]. The incidence of T2D was 58% lower (95% CI: 48–66%) among subjects who were assigned to the lifestyle-modification program (with a goal of at least a 7% weight loss of the baseline body weight) than those in the placebo group [33]. Additionally, weight loss among subjects in the lifestyle-modification program was significantly and independently associated with reductions in blood glucose from pre-diabetic to normal levels [34]. Weight loss was also associated with long-term benefit in a follow-up study of the DPP program, which found that the 10-year cumulative incidence of T2D among participants in the lifestyle-modification program was lower compared with those treated with metformin or in the placebo group [35].

The risk of T2D associated with each BMI level was estimated, adjusting for other covariates. To assess the impact of the other covariates, we estimated an unadjusted logistic regression model (with BMI level as the only covariate); the resulting ORs were 1.9 (95%CI: 1.7–2.0) for overweight, 3.7 (3.4–4.0) for Obesity Class I, 6.7 (6.2–7.4) for Obesity Class II, and 12.7 (11.6–14.0) for Obesity Class III (all p-values < 0.01). The unadjusted ORs were slightly higher than the adjusted ORs. This implies that some factors, such as age, are associated with both increased BMI and increased risk of T2D, but the impact of these factors on the association between BMI and risk of T2D is limited.

Besides the association between BMI and risk of T2D, our study also revealed other interesting findings. For example, the BMI value considered in our study was the last one observed during the 12 months before the index date, which tended to be closer to the index date among case individuals than control individuals (9.0 vs. 80.3 days, p < 0.01). This implies that individuals (and/or their physicians) may have started monitoring their BMI (and probably other clinical biomarkers) more frequently when there were indications that they may be at risk of developing T2D.

The risk of developing T2D for individuals who were overweight or obese was about 1.5–5 times higher than for individuals with normal BMI, as estimated in our study. This demonstrates the importance of continuous weight management, which not only can reduce the disease burden of obesity but also may prevent further progression to T2D. Weight management is particularly important for people with severe obesity, who were disproportionally at higher risk of developing T2D than individuals with less severe obesity. Physicians should regularly monitor the weight of their patients with obesity.

Our results should be interpreted in light of the study’s limitations. First, and foremost, the use of a retrospective cohort design prevented us from understanding the causal effect of BMI on the risk of developing T2D. Second, although cases and controls were matched on broad demographic and clinical characteristics (except for BMI measures) while selecting them from the Geisinger Health System database, the distribution of demographic and baseline clinical characteristics between cases and controls in our final analytic sample were not balanced. We addressed this imbalance by adjusting for a detailed list of confounding factors, but the potential for unmeasured, and unadjusted, confounding in baseline characteristics may have remained. Another limitation is that Geisinger Health System data cannot capture health services provided outside of the system. Without available enrollment data, we considered individuals to have continuously received care in the Geisinger Health System during the 12-month pre-index period if they had activity recorded in 365 or more days before the index date. This approach is unlikely, we feel, to introduce substantial bias since there is no evidence that the likelihood for individuals to seek care outside of the Geisinger system was correlated with T2D or obesity (personal communication, Christopher Still and Thomas Graf). A similar approach has been used in previous retrospective electronic health records database studies [36,37]. Significant clinical and/or body size differences between people with and without BMI measures may also serve as another potential source of bias. Finally, the findings are based on data from a single integrated health system caring for individuals in Pennsylvania and may not be generalizable to larger populations and to other regions in the US.

## Conclusion

Using a large cohort of individuals with detailed electronic health records, we were able to show that not only is BMI strongly and independently associated with the risk of being diagnosed with T2D, but also that the magnitude of this positive association is larger for higher BMI values. Further research on the association between BMI and the risk of developing T2D should include the time to the incident T2D diagnosis and, if data are available, account for individuals’ pre-diabetic status and the timing and duration of obesity.

## Appendix. Sample Selection Criteria

Case and control individuals needed to satisfy the following criteria:

• During their 12-month pre-index period they had to have:

 ◦ Continuously received care in the Geisinger Health System (defined by having encounters that occurred 365 or more days before the index date)

 ◦ Not be pregnant

 ◦ Not had bariatric surgery

 ◦ At least one valid BMI measurement

• Cases and controls were excluded if they had:

 ◦ Any BMI measurement <18.5 kg/m^2^ during the 12-month pre-index period

 ◦ Any evidence of type 1 diabetes, during the study period, defined by

  ▪ ICD-9-CM diagnosis codes of 250.x1 or 250.x3

  ▪ A medication order for insulin without a diabetes diagnosis code

## Abbreviations

BMI: Body mass index; CI: Confidence interval; DPP: Diabetes Prevention Program; OR: Odds ratio; T2D: Type 2 diabetes; US: United States.

## Competing interests

This research was supported by Novo Nordisk. Neil Wintfeld, Jakob Langer, and Mette Hammer are employees of Novo Nordisk. Mette Hammer and Jakob Langer are also stockholders of Novo Nordisk. Michael Ganz, Qian Li, and Veronica Alas are employees of Evidera, which has received research funds from Novo Nordisk to conduct this study.

## Authors’ contributions

MLG, NW, and QL designed the study. The data analysis and interpretation of data was done by QL. Manuscript writing and revisions performed by MLG, NW, QL, VA, JL, and MH. MLG, NW, MH decided to submit the paper for publication. All authors read and approved the manuscript.

## References

[B1] Tight blood pressure control and risk of macrovascular and microvascular complications in type 2 diabetes: UKPDS 38. UK Prospective Diabetes Study Group BMJ 1998 317 7160 703 713 10.1136/bmj.317.7160.703 9732337 PMC28659

[B2] Beckman JA Creager MA Libby P Diabetes and atherosclerosis: epidemiology, pathophysiology, and management JAMA 2002 287 19 2570 2581 10.1001/jama.287.19.2570 12020339

[B3] Heron M Hoyert DL Murphy SL Xu J Kochanek KD Tejada-Vera B Deaths: final data for 2006 Natl Vital Stat Rep 2009 57 14 1 134 19788058

[B4] Riddle MC Glycemic control and cardiovascular mortality Curr Opin Endocrinol Diabetes Obes 2011 18 2 104 109 10.1097/MED.0b013e3283446b7e 21522000

[B5] Economic costs of diabetes in the U.S. in 2012 Diabetes Care 2013 36 4 1033 1046 23468086 10.2337/dc12-2625 PMC3609540

[B6] National Institute of Diabetes and Digestive and Kidney Diseases National Diabetes Statistics, 2007 Fact Sheet 2008 Bethesda, MD: U.S. Department of Health and Human Services, National Institutes of Health

[B7] Centers for Disease Control and Prevention National diabetes fact sheet: national estimates and general information on diabetes and prediabetes in the United States, 2011 2011 Atlanta, GA: U.S. Department of Health and Human Services, Centers for Disease Control and Prevention

[B8] Olokoba AB Obateru OA Olokoba LB Type 2 diabetes mellitus: a review of current trends Oman Med J 2012 27 4 269 273 10.5001/omj.2012.68 23071876 PMC3464757

[B9] Garber AJ Obesity and type 2 diabetes: which patients are at risk? Diabetes Obes Metab 2012 14 5 399 408 10.1111/j.1463-1326.2011.01536.x 22074144

[B10] Flegal KM Carroll MD Kit BK Ogden CL Prevalence of obesity and trends in the distribution of body mass index among US adults, 1999–2010 JAMA 2012 307 5 491 497 10.1001/jama.2012.39 22253363

[B11] Cawley J Meyerhoefer C The medical care costs of obesity: an instrumental variables approach J Health Econ 2012 31 1 219 230 10.1016/j.jhealeco.2011.10.003 22094013

[B12] Wang Y Beydoun MA Liang L Caballero B Kumanyika SK Will all Americans become overweight or obese? estimating the progression and cost of the US obesity epidemic Obesity (Silver Spring) 2008 16 10 2323 2330 10.1038/oby.2008.351 18719634

[B13] Kodama S Horikawa C Fujihara K Heianza Y Hirasawa R Yachi Y Sugawara A Tanaka S Shimano H Iida KT Saito K Sone H Comparisons of the strength of associations with future type 2 diabetes risk among anthropometric obesity indicators, including waist-to-height ratio: a meta-analysis Am J Epidemiol 2012 176 11 959 969 10.1093/aje/kws172 23144362

[B14] Carey VJ Walters EE Colditz GA Solomon CG Willett WC Rosner BA Speizer FE Manson JE Body fat distribution and risk of non-insulin-dependent diabetes mellitus in women, The Nurses' Health Study Am J Epidemiol 1997 145 7 614 619 10.1093/oxfordjournals.aje.a009158 9098178

[B15] Shai I Jiang R Manson JE Stampfer MJ Willett WC Colditz GA Hu FB Ethnicity, obesity, and risk of type 2 diabetes in women: a 20-year follow-up study Diabetes Care 2006 29 7 1585 1590 10.2337/dc06-0057 16801583

[B16] Abdullah A Stoelwinder J Shortreed S Wolfe R Stevenson C Walls H de Courten M Peeters A The duration of obesity and the risk of type 2 diabetes Public Health Nutr 2011 14 1 119 126 10.1017/S1368980010001813 20587115

[B17] Wang H Shara NM Calhoun D Umans JG Lee ET Howard BV Incidence rates and predictors of diabetes in those with prediabetes: the Strong Heart Study Diabetes Metab Res Rev 2010 26 5 378 385 10.1002/dmrr.1089 20578203 PMC2897954

[B18] Zindah M Belbeisi A Walke H Mokdad AH Obesity and diabetes in Jordan: findings from the behavioral risk factor surveillance system, 2004 Prev Chronic Dis 2008 5 1 A17 18082006 PMC2248793

[B19] Krishnan SRL Djousse L Cupples LA Palmer JR Overall and central obesity and risk of type 2 diabetes in U.S. black women Obesity (Silver Spring) 2007 15 7 1860 1866 10.1038/oby.2007.220 17636105

[B20] Mokdad AH Ford ES Bowman BA Dietz WH Vinicor F Bales VS Marks JS Prevalence of obesity, diabetes, and obesity-related health risk factors, 2001 JAMA 2003 289 1 76 79 12503980 10.1001/jama.289.1.76

[B21] Velasco Mondragon HE Charlton RW Peart T Burguete-Garcia AI Hernandez-Avila M Hsueh WC Diabetes risk assessment in Mexicans and Mexican Americans: effects of parental history of diabetes are modified by adiposity level Diabetes Care 2010 33 10 2260 2265 10.2337/dc10-0992 20628089 PMC2945171

[B22] Asche C Nelson R McAdam-Marx C Jhaveri M Ye X Predictors of oral bisphosphonate prescriptions in post-menopausal women with osteoporosis in a real-world setting in the USA Osteoporos Int 2010 21 8 1427 1436 10.1007/s00198-009-1079-7 19798459 PMC2895897

[B23] Herout PM Harshaw Q Phatak H Saka G McNeill A Wu D Sazonov V DeSagun R Shirani J Impact of worsening renal function during hospital admission on resource utilization in patients with heart failure Am J Cardiol 2010 106 8 1139 1145 10.1016/j.amjcard.2010.06.026 20920654

[B24] Nordstrom BL Kachroo S Fraeman KH Nutescu EA Schein JR Fisher A Bookhart BK Warfarin prophylaxis in patients after total knee or hip arthroplasty–international normalized ratio patterns and venous thromboembolism Curr Med Res Opin 2011 27 10 1973 1985 10.1185/03007995.2011.614938 21919556

[B25] Stecker MM The EEG as an independent indicator of mortality and healthcare utilization Clin Neurophysiol 2009 120 10 1777 1781 10.1016/j.clinph.2009.07.041 19699144

[B26] Wu EQ Zhou S Yu A Lu M Sharma H Gill J Graf T Outcomes associated with insulin therapy disruption after hospital discharge among patients with type 2 diabetes mellitus who had used insulin before and during hospitalization Endocr Pract 2012 18 5 651 659 10.4158/EP11314.OR 22440990

[B27] United States Department of Labor, Bureau of Labor Statistics Consumer Price Indexhttp://www.bls.gov/cpi/

[B28] Kleinman LC Norton EC What's the Risk? A simple approach for estimating adjusted risk measures from nonlinear models including logistic regression Health Serv Res 2009 44 1 288 302 10.1111/j.1475-6773.2008.00900.x 18793213 PMC2669627

[B29] Efron B TR: An Introduction to the Bootstrap 1993 New York: Chapman and Hall

[B30] Rothman KJ Epidemiology: An Introduction 2002 Oxford University press: New York

[B31] Burkhauser RV Cawley J Beyond BMI: the value of more accurate measures of fatness and obesity in social science research J Health Econ 2008 27 2 519 529 10.1016/j.jhealeco.2007.05.005 18166236

[B32] Plankey MW Stevens J Flegal KM Rust PF Prediction equations do not eliminate systematic error in self-reported body mass index Obes Res 1997 5 4 308 314 10.1002/j.1550-8528.1997.tb00556.x 9285836

[B33] Knowler WC Barrett-Connor E Fowler SE Hamman RF Lachin JM Walker EA Nathan DM Reduction in the incidence of type 2 diabetes with lifestyle intervention or metformin N Engl J Med 2002 346 6 393 403 11832527 10.1056/NEJMoa012512 PMC1370926

[B34] Perreault L Kahn SE Christophi CA Knowler WC Hamman RF Regression from pre-diabetes to normal glucose regulation in the diabetes prevention program Diabetes Care 2009 32 9 1583 1588 10.2337/dc09-0523 19587364 PMC2732165

[B35] Knowler WC Fowler SE Hamman RF Christophi CA Hoffman HJ Brenneman AT Brown-Friday JO Goldberg R Venditti E Nathan DM 10-year follow-up of diabetes incidence and weight loss in the Diabetes Prevention Program Outcomes Study Lancet 2009 374 9702 1677 1686 19878986 10.1016/S0140-6736(09)61457-4 PMC3135022

[B36] Pawaskar M Li Q Hoogwerf BJ Reynolds MW Faries D Engelman W Bruhn D Bergenstal RM Metabolic outcomes of matched patient populations initiating exenatide BID vs. insulin glargine in an ambulatory care setting Diabetes Obes Metab 2012 14 7 626 633 10.1111/j.1463-1326.2012.01581.x 22321776

[B37] Wu N Aagren M Boulanger L Friedman M Wilkey K Assessing achievement and maintenance of glycemic control by patients initiating basal insulin Curr Med Res Opin 2012 28 10 1647 1656 10.1185/03007995.2012.722989 22937724

